# First-Principles Study on Evolution of Magnetic Domain in Two-Dimensional BaTiO_3_ Ultrathin Film Doped with Co under Electric Field

**DOI:** 10.3390/nano14070586

**Published:** 2024-03-27

**Authors:** Haigen Gao, Yu Tang, Qilong Liao, Xiangyu Zhao, Bing Wang

**Affiliations:** School of Electrical and Information Engineering, Panzhihua University, Panzhihua 617000, China; gaohaigen@pzhu.edu.cn (H.G.); tangyu@pzhu.edu.cn (Y.T.); liaoqilongpzh@pzhu.edu.cn (Q.L.)

**Keywords:** first principles, two-dimensional BaTiO_3_, Co substitution, mechanism of nonmagnetic characteristics, ferromagnetic behavior under electric field, magnetoelectric coupling

## Abstract

The magnetization mechanism of Co-doped BaTiO_3_ ultrathin films is a subject of debate, which results in difficulties with the design of new multiferroics based on BaTiO_3_ matrixes. With the aid of a first-principles approach, it was observed that when the interstitial site and Ti vacancy were filled with Co, the configuration behaved in a nonmagnetic manner, indicating the significance of the Co content. Moreover, in the case of Co substituting two neighboring Ti atoms, when a direct current field was applied in the [100] direction, the magnetic domains excluding those in the [100], [010], and [001] directions were directed away. Further, the magnetoelectric constant was evaluated at ~449.3 mV/cmOe, showing strong magnetoelectric coupling at room temperature. Clearly, our study indicates that strict control of Ba, Ti, O, and Co stoichiometry can induce an electric and magnetic field conversion in two-dimensional BaTiO_3_ and may provide a new candidate for single-phase multiferroics for application in next-generation multifunctional devices.

## 1. Introduction

Barium titanate (BaTiO_3_) is an archetypal ferroelectric perovskite and has been widely applied in multiferroic composites, electronic devices, etc., owing to its high spontaneous polarization [1,2,3,4,5]. Different from the well-known perovskite oxides BiFeO_3_ and BiMnO_3_ [6,7], its spontaneous polarization mainly originates from displacement between Ti and O [8,9,10]. In theory, substituting Ti or Ba with ferromagnetic atoms can achieve the coexistence of ferromagnetic and ferroelectric orders [11,12,13], which may resolve issues of weak magnetoelectric (ME) coupling in single-phase multiferroics, such as BiFeO_3_, at room temperature. However, most current investigations lack a detailed description of this phenomenon, including interactions between ferroic orders. In 2019, Gao et al. [14] reported that Fe substituting Ti and Ba vacancies can induce a magnetic and electric field conversion in two-dimensional BaTiO_3_, which indicates that ferromagnetic doping, with the aid of external fields, is available for designing new multiferroics from BaTiO_3_ matrixes with the ability to select domain orientations. Although the ferroelectric behavior of BaTiO_3_ has been clarified, the ferromagnetic characteristics are still under study; hence, to explore the evolution of the ferromagnetic domain under an electric field, density functional theory (DFT) was employed to shed light on the relationship between ferromagnetic orders and external fields. 

As our previous study showed, multiferroic behavior was observed in two-dimensional BaTiO_3_ with Fe substituting Ti, where the origin of ferromagnetism resulted from not only the dopant but also elemental spontaneous polarization [14]. However, whether it can be utilized to interpret all phenomena appearing in these ferromagnetic replacements needs to be confirmed. For instance, a Co-doped BaTiO_3_ ultrathin film grown by Ponath et al. showed a nonmagnetic signal [15], while a film prepared by Lin et al. demonstrated a hysteresis loop [16]. This suggests that a debate exists in BaTiO_3_ magnetization with Co substituting Ti. Consequently, when seeking a new single-phase system, it must be made clear that BaTiO_3_ exhibits great potential for application as a high-performance multiferroic [8,14]. To date, Fe substitution accounts for the largest percentage of performed investigations [17,18,19,20,21], where Mn, Cr, Ni, etc., introduce a magnetic moment into BaTiO_3_. However, most studies state that when the dopant content increases, the ferromagnetism is enhanced, ferroelectricity is improved, and there is further clarification on the interaction between ferroic orders [22,23,24]. Both Ba and Ti replacements were carried out in the previous studies and showed different physical properties. In the former, structural disorder appeared and reduced the tolerance factor and tetragonality ratio of BaTiO_3_ [25]. Thus, to maintain structural stability, Ti substitution is a better choice to introduce ME coupling in BaTiO_3_; however, it sometimes produces weak ME coupling, indicating that the origin of multiferroic properties may result from metal diversity. To obtain a universal mechanism for describing ME coupling induced by different metals, magnetization induced via Co was studied, and its ferromagnetic behavior under an electric field was investigated. 

The BaTiO_3_ epitaxial structure growing process is complicated because Co might occupy the interstitial spaces, the vacancies, and the interstitial and vacancy as well as substitutional sites, which results in different magnetization mechanisms. To determine possible chemical components, molecular dynamics was employed to simulate conditions of rich Co and poor Ti. Moreover, an electric field was applied to explore the evolution of the ferromagnetic domains, which showed a superior multiferroic property in two-dimensional BaTiO_3_ at room temperature, compared with that with Fe occupying Ti sites [14]. Lee et al. experimentally showed that ferroelectricity is sustained in the BaTiO_3_ film as thin as two unit cells [26]. Hence, to fundamentally understand the mechanism of ME coupling, the minimum thickness of two-dimensional BaTiO_3_ was set as *m* = 2 unit cells. Taking phase transition induced by substitution into account, the amount of Co must be less than that making the band gap of two-dimensional BaTiO_3_ disappear. And in this study, the structural stability, magnetization, magnetic behavior under an electric field, and magnetic and electric field conversion are studied; it exhibits a great prospect for BaTiO_3_ application in multifunctional devices and will induce further experimental investigations. 

## 2. Methods

In this study, the thicknesses of BaTiO_3_ (001) and (100) ultrathin film models *m* were defined as two unit cells. The magnetic moment, spontaneous polarization, and their changes under external fields, etc., were studied using the first-principles plane-wave pseudopotential method based on DFT as implemented in the Vienna ab initio simulation package (VASP) [27]. Projector augmented-wave pseudopotential with generalized gradient approximation (GGA) of the PBEsol functional was used in all calculations [28]. The semi-core Ba 5*s* and 5*p* and Ti 3*s* and 3*p* states were treated as valence states. The plane-wave energy cutoff was set at 500 eV, and the residual forces were less than 0.01 eV/Å in the geometry optimization using the conjugate gradient algorithm [29,30]. The simulation model contained five layers along the *z*-axis (or *x*-axis) and a vacuum possessing a capacity of 4 BaTiO_3_ unit cells above the top surface (or from the left surface) and below the bottom surface (or relative to the right surface), respectively, and the dimension in the *xy*-plane (or *yz*-plane) was set at 2 × 2, 2 × 3, 4 × 2, 4 × 3, and 6 × 2, respectively. The corresponding Brillouin zone integration was calculated using a *k*-point mesh of 7 × 7 × 1, 7 × 7 × 1, 5 × 7 × 1, 5 × 5 × 1, and 3 × 7 × 1 automatically generated using the Monkhorst–Pack method [31]. To estimate spontaneous polarization, the berry-phase and density functional perturbation theory (DPFT) methods were implemented and VASP was employed, and the corresponding results were calculated to be 28.9 µC/cm^2^ and 29.2 µC/cm^2^, in good agreement with the experimental value of 26.l µC/cm^2^ and a previous prediction of 29.9 µC/cm^2^ [14,32,33,34]. To evaluate the spontaneous polarization per unit cell, DPFT was adopted, and the spontaneous polarization was assumed to map onto the center Ti atom in a unit cell. To confirm electric and magnetic field conversion, an electric field was applied in the [001] direction, which can be implemented in the VASP 5.4.4 software by setting IDIOPLE = TRUE. Moreover, we adjusted the parameter of EFIELD as described by Yeh et al. [35], and a ~32 Å vacuum layer was built in this direction. The electric field ranged from 0 MV/m to 800 MV/m.

## 3. Results and Discussions

### 3.1. Structural Stability and Magnetic Properties of BaTiO_3_ Ultrathin Film 

**Figure 1:**
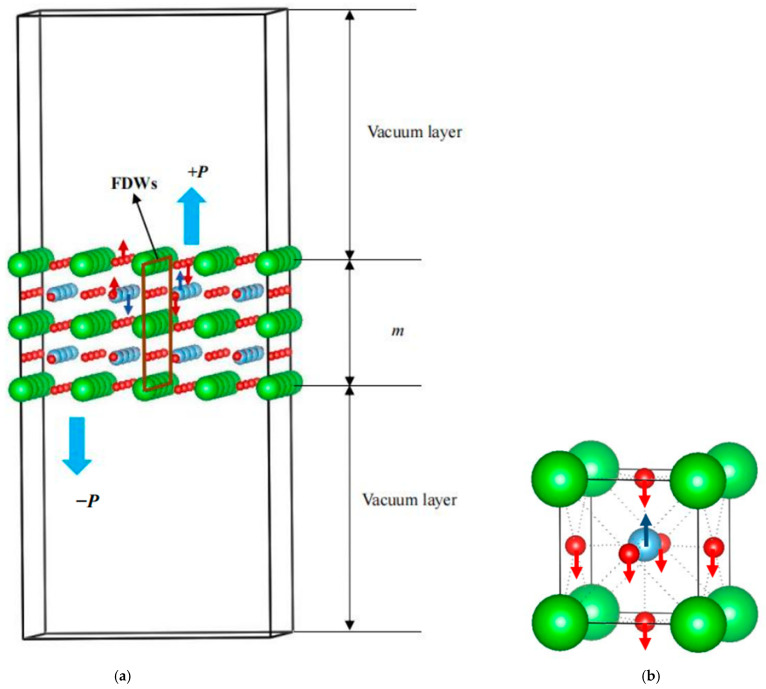
Simulation model of a two-dimensional BaTiO_3_ (001) ultrathin film with perfect crystal structure and thickness *m* = 2; the Ba, Ti, and O atoms are represented as green, blue, and red colors; the FDWs are used to denote the ferroelectric domain wall and comprise O/Ba atoms; the red and blue arrows show the movement of Ti and O atoms. (**a**) Simulation model of a two-dimensional BaTiO_3_ (001) ultrathin film; (**b**) unit cell of BaTiO_3_.

As clarified above, BaTiO_3_ epitaxial structure magnetization is still a subject of debate, which brings difficulty in designing new multiferroics based on two-dimensional BaTiO_3_; thus, to clarify the corresponding mechanism, simulation models of BaTiO_3_ (100) and (001) ultrathin films were built. Figure 1 is the configuration with BaO termination and shows an anti-ferroelectric domain, which was confirmed to be available in our previous studies [8,14]. The unit cell in two-dimensional BaTiO_3_ is in the tetragonal state, as shown in Figure 1b, where the movement of Ti and O is represented using the red and blue arrows and results in spontaneous polarization. A similar structural feature has been observed in a 2.5-unit-cell-thick BaTiO_3_ ultrathin film grown on a Ge (001) substrate [36]. +*P* and −*P* were used to represent the up and down spontaneous polarization perpendicular to the surface, where +*P* is the spontaneous polarization oriented out of the top surface and −*P* is that orientated into the top surface. In all calculations, the plane-wave energy cutoff was set at 500 eV, and the optimized lattice parameters were evaluated to be *a* = *b* = 3.979 Å/*c* = 4.0304 Å, nearly equal to experimental data of *a* = *b* = 3.997 Å/*c* = 4.0314 Å [37]. Here, it is worth stating that in a previous study, the structure was optimized with a fixed volume, and the geometric parameters *a* = *b* = 3.979 Å/*c* = 4.064 Å were calculated using a cutoff of 520 eV, which approximated the theoretical values *a* = *b* = 3.980 Å/*c* = 4.076 Å [38]. In addition, the parameters utilized by Ponath et al. were also adopted in this study, where the magnetic moment of Co was calculated to be 0.92 μ_B_ in the case of the low-spin state, agreeing well with the referenced study [8]. Clearly, the selected methodology is reasonable for implementation in this study. For simplicity, only the charge equilibrium was considered here.

To date, the view of whether ferromagnetism can be induced in BaTiO_3_ ultrathin film with the usage of Co substituting Ti is still contradictory. The possible positions of Co in the lattice were studied to provide insights into this phenomenon. Both the (010) and (001) configurations were considered. They showed similar phenomena of stability, spin–spirals, and ferroelectricity, corresponding to the free-standing states. Here, the (001) configuration was adopted as an example for exploring the origin of the magnetic signal. First, the energetic stability was evaluated with the help of the formation energy, defined as follows [39,40]:

(1) $$E_{f}^{V}=E_{tot}(ref+V)-E_{tot}(ref)+\mu_{A}.$$

where *E_f_^V^* is the formation energy of vacancy; *E_tot_* (*ref*) and *E_tot_* (*ref + V*) are the total energies of the perfectly crystalline structures without and with vacancies, respectively; and *µ_A_* is the chemical potential of isolated atom A removed or added to form a vacancy or interstitial defect, and calculated based on bulk Co. Analogically, the formation energy of the dopant incorporating into vacancies and interstitial sites was deduced from Equations (2) and (3) [39,40]:

(2) $$E_{f}^{V+A}=E_{tot}(ref+V+A)-E_{tot}(ref+V)-\mu_{A}.$$

(3) $$E_{f}^{A}=E_{tot}(ref+A)-E_{tot}(ref)-\mu_{A}.$$

where *E^V+A^_f_* and *E^A^_f_* are the formation energies of the dopant atom incorporating into vacancies and interstitial sites, *E_tot_* (*ref + V + A*) and *E_tot_* (*ref + A*) are the total energies of the system with the dopant atom incorporating into vacancies and interstitial sites, and *E_tot_* (*ref + V*) and *E_tot_* (*ref*) are the total energy of the system with and without vacancies. 

Taking structural stability into account, only Ti substitutions were implemented, owing to the large crystal distortion induced upon replacing Ba, which reduced the tolerance factor and tetragonal ratio [25]. Table 1 is the formation energy of Co occupying different sites. These selected positions are interstitial spaces, vacancies, and the interstitial and vacancy sites. For vacancies, the formation energy is −3.26 eV, which, when below zero, indicates energetic stability. In the case of Co diffusing into vacancies and interstitial sites simultaneously, the formation energy varies with the distance between the interstitial and vacant Co (interstitial and vacant Co means the Co in the interstitial site and vacancy, respectively). For instance, when the distance is 0.5 unit cells, the formation energy is −2.08 eV; when increasing the distance to 1.5 unit cells, it is calculated at −0.64 eV; then, even if the distance is enhanced constantly, the formation energy remains around −0.6 eV. In this study, the enthalpy of formation for two-dimensional BaTiO_3_ was calculated, which was defined as the difference in total energy of two-dimensional BaTiO_3_ and the energies of its constituent elements in their stable states [41]:

(4) $$\begin{array}{l} \Delta_{f}E({Ba}_{x}{Ti}_{y}O_{z}{Co}_{m})\\ \;\;\;\;\;\;\;=E({Ba}_{x}{Ti}_{y}O_{z}{Co}_{m})-\frac{x}{x+y+z+m}E(Ba)-\frac{y}{x+y+z+m}E(Ti)-\frac{z}{x+y+z+m}E(O)\\ \;\;\;\;\;\;\;-\frac{m}{x+y+z+m}E(Co) \end{array}$$

where *E* (${Ba}_{x}{Ti}_{y}O_{z}{Co}_{m}$) is the total energy of the compound, and *E* (Ba), *E* (Ti), *E* (O), and *E* (Co) are the total energies of the pure elements in their stable structures.

Metallic Co has two different crystal structures: one is hexagonal close-packed (HCP), and the other is face-centered cubic (FCC). At room temperature, Co exists in the HCP structure, and when the temperature exceeds 450 °C, it exists in the FCC configuration. By calculation, the corresponding total energies of pure Ti elements in their stable structures are −7.83 eV and −7.81 eV, respectively. Clearly, they approximate each other. Thus, the enthalpy of the formation of two-dimensional BaTiO_3_ is ~5.47 eV, showing energetic stability. Interestingly, the total magnetic moments are nil when the ratio of interstitial and vacant Co is 1:1, indicating the possibility of nonmagnetic configurations grown with experimental technology. Moreover, for pure interstitial Co, the formation energy was evaluated to be −7.77 eV, lower than that of the vacancies, suggesting that Co is more readily incorporated into the atomic interval, and the corresponding magnetic moment was calculated to be 1.44 μ_B_. As a comparison, that of Co in vacancies was estimated at 1.00 μ_B_. That is to say, both interstitial and vacant Co can induce a spin–spiral order in BaTiO_3_. In addition, when Co is in the interstitial sites nearest to vacancies (i.e., the vacancy is empty), the total magnetic moment is 3.96 μ_B_, but this configuration has a large crystal distortion. To provide a full picture of Co substitution, the following cases were considered: First, Co in Ti sites exceeds that occupying interstitial spaces; second, vacant Co exceeds that occupying interstitial spaces. When the atomic number ratio of interstitial and vacant Co is 2:1 or 1:2, the magnetic moments are approximately 0.99 μ_B_ and 0.98 μ_B_. This shows that when interstitial Co forms, the total magnetic moment decreases. It also indicates that an appropriate preparation method is crucial to avoid Co entering into interstitial spaces. 

Our previous study showed that foreign atoms readily diffuse into vacancies relative to interstitial spaces, which can be evaluated using the trapping energy, which is expressed by Equation (5) [42,43]:

(5) $$E_{trap}=E_{tot}(ref+nA,V)-E_{tot}(ref+(n-1)A,V)-E_{tot}(ref+A)+E_{tot}(ref)$$

where *E_tot_* (*ref + nA*,*V*) and *E_tot_* (*ref + A*) are the total energies of perfect crystals with n foreign atoms in a single vacancy and with one foreign atom in an interstitial site, respectively.

The negative trapping energy indicates that a foreign atom trapped in a vacancy is more stable than when it is located at an interstitial site; in other words, this might mean that when the trapping energy is positive, foreign atoms will diffuse into interstitial spaces. Here, the trapping energy of Co is 5.69 eV, indicating that Co will occupy an interstitial site rather than a vacancy, which was confirmed using the molecular dynamics study. Then, the energy of the formation of the O vacancy was studied. First, more than one Co atom was incorporated into interstitial spaces to remove the nearest O atom to form a vacancy, but when two Co were added, the corresponding trapping energy was 2.39 eV. This shows that the maximum number of Co in an interstitial site is one. Assuming that an O vacancy is formed, the corresponding formation energy is 8.87 eV and positive, showing an energetically unstable structure. That is to say, the growing technology or heat treatment may induce the O vacancy, i.e., when the atmosphere of heat treatment is oxygen deficient, an O vacancy will form. Clearly, our study provides supplementary insight into the formation of O vacancies in BaTiO_3_ ultrathin films doped with Co and suggests that O vacancy formation is difficult.

Generally, our calculations illustrate a clue to the origin of the magnetic properties in BaTiO_3_ ultrathin films doped with Co, where interstitial Co is readily formed, resulting in the disappearance of magnetic signals. This indicates that avoiding interstitial Co is crucial to induce a spin–spiral order in Co-doped BaTiO_3_ ultrathin films. Although Pontal et al. provided an interpretation of nonmagnetic signals with the aid of DFT, it is insufficient because a detailed study on the formation of O vacancies, which is the main factor resulting in the antiferromagnetic domain, is absent [15].

### 3.2. Evolution of Magnetic Behavior under Electric Field

**Figure 2:**
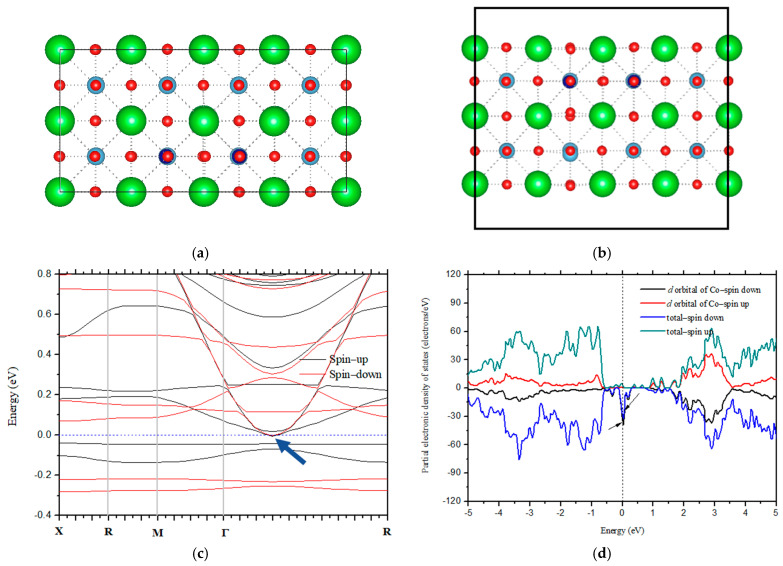
Optimized geometric and electronic structures of two-dimensional BaTiO_3_ (001) doped with a ~2.08% atomic concentration of Co in the Ti sites; (**a**) vertical and (**b**) front views of geometric structure, (**c**) band structure, and (**d**) total and partial electronic density states. In the geometric structures, the dotted lines denotes the chemical bonds between atoms; in the electronic structures, they represent the Fermi level, and Fermi energy are set to 0 eV. The arrow points to the the band gap that is zero.

It is well known that electronic properties are dependent on dopant content. Thus, the band structures were calculated to determine the magnitude of Co required to maintain BaTiO_3_ behavior in the manner of a semiconductor, as shown in Figure 2. Figure 2a,b are the optimized geometric structures of two-dimensional BaTiO_3_ with a 2.08% atomic concentration of Co substituting Ti, which shows that this configuration retains the tetragonal state. That is because the lattice parameters are *a = b =* 3.970 Å*/c* = 4.005 Å and *α = β = γ* = 90^o^. In a previous study, the electronic structures of pure two-dimensional BaTiO_3_ ultrathin films were discussed, and it was shown that it is a semiconductor with a Kahn–Sham gap of ~2.0 eV [8]. Here, it can be seen that when the atomic concentration (the number of Co vs. total atoms in the supercell) reaches 2.08%, the band gap of BaTiO_3_ disappears, as shown in Figure 2c. Moreover, it is in good agreement with the experimental report, where the band gap is narrowed with enhanced Co [44]. To further understand the Fermi level crossing induced via Co-doping, the partial electronic density of different states was also plotted. Figure 2d shows that the *d* orbits of Co cross the Fermi level. When the magnitude of Co increases to ~2.08%, the *d* orbits of Co fill the band gap and cause the two-dimensional BaTiO_3_ to behave in the manner of a conductor. Clearly, Co must be strictly controlled, particularly for the electrical adjustment of magnetic behavior through applying an electric field. The proportioning of Co/Ba/Ti/O must be no more than their stoichiometric ratio obtained from the calculation. Due to the tendency of Co diffusing into the interstitial site to cause nonmagnetic behavior, the ratio of Co/Ti/O/Ba must be evaluated precisely, especially Co and Ti. When Co exceeds the experimental stoichiometry, it diffuses into the interstitial space. Clearly, Co must be uniformly distributed to prevent local enrichment. In addition, the Co concentration must be controlled to maintain its semiconductor characteristics. In the doping process, the heat treatment temperature is high because dispersion is inferior when the temperature is low [45], which may result in a local Co accumulation. 

**Figure 3:**
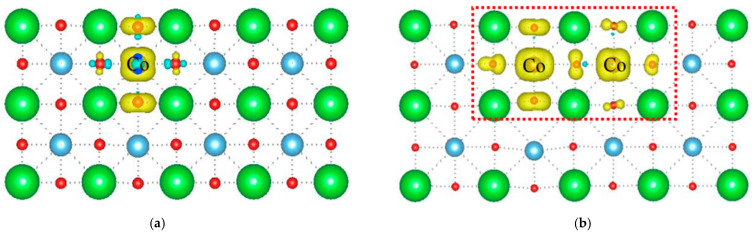
Spin-polarized charge density of a two-dimensional BaTiO_3_ (001) ultrathin film with Co in Ti sites: (**a**) one Co atom; (**b**) two neighboring Co atoms. The asymmetric spin-polarized charge density caused by neighboring substitution is denoted using the red box. The dotted lines represent the chemical bonds between atoms. And the red box represent the distribution of spin-polarized charge densities.

**Figure 4:**
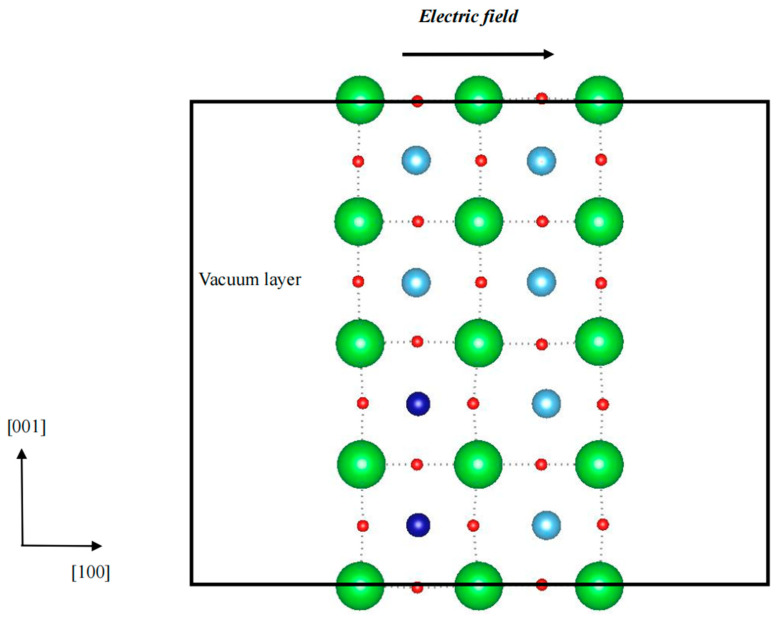
Simulation model of a two-dimensional BaTiO_3_ (100) ultrathin film with a 2 × 3 × 4 dimension; the direction of the electric field is denoted using the arrow. The total vacuum layer is ~32 Å from left and right surfaces. The Co, Ba, Ti, and O atoms are represented as the deep blue, green, blue, and red colors. The dotted lines denote the BaTiO_3_ unit cell.

**Figure 5:**
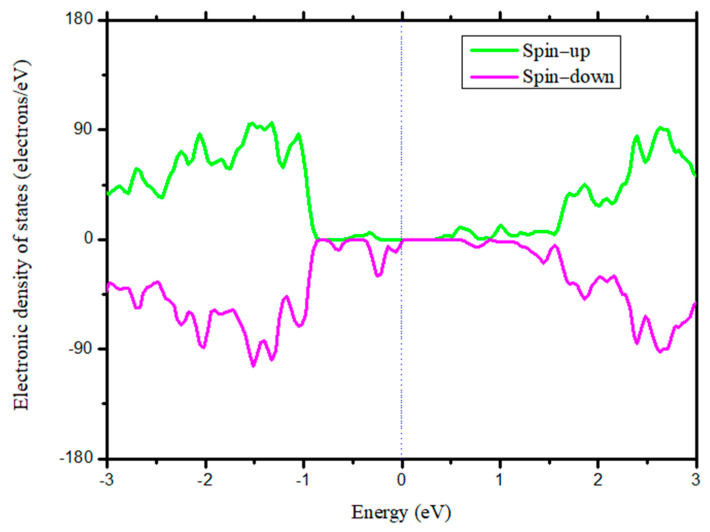
Electronic density of states of two-dimensional BaTiO_3_ (001) ultrathin film with a 2 × 3 × 4 dimension.

To provide an insight into the relationship between the dopant and spin–spiral order, the magnetic moment as a function of Co content was studied. It was found that the magnetic moment increases linearly with enhanced Co content in the case of Co substituting Ti with at least two unit-cell intervals. For instance, when the number of Co atoms is 1, the magnetic moment is 1.00 μ_B_; upon increasing it to 3, the magnetic moment is enhanced to 3.00 μ_B_; as a comparison, Co occupying neighboring Ti sites introduces a larger magnetic moment. For the neighboring substitution of two Ti atoms, the total magnetic moment of the supercell is evaluated at 4.00 μ_B_, and the three Cr replacements show a 5.00 μ_B_ magnetic moment. In comparison, seven Co atoms induce a 14.97 μ_B_ magnetic moment. Similar to neighboring substitution using Fe, neighboring Co replacement indicates coupling between dopants. The spin-polarized charge density was plotted to explore these interactions. Figure 3a,b are the spin-polarized charge densities of configurations with one and two neighboring Co atoms. They show an asymmetric distribution of spin-polarized charge density in the neighboring substitution, indicating that a spontaneous polarization is formed in the [100] direction, as denoted in the red box. The spin-polarized charge density of O atoms in the doped cell is also asymmetric, suggesting that the spin states of electrons in the neighboring Co differ from each other. In the case of one Co in a Ti site in the supercell, it demonstrates a symmetric distribution of spin-polarized charge density, which suggests that the substitution induces no spontaneous polarization. That is to say, the neighboring replacement of Ti can introduce an ME coupling, which will induce a magnetization with an external electric field or an electrical polarization upon applying an external magnetic field; hence, it was adopted to study the electrical control of the magnetic behavior of BaTiO_3_ ultrathin films. Taking the electronic property as a function of Co contents into account, a supercell with a dimension of 2 × 3 × 4 was built, comprising 144 atoms, as shown in Figure 4. Its electronic density of states was calculated and is plotted in Figure 5, showing characteristics of semiconductors. Thus, the spontaneous polarization was calculated using DFPT to analyze this phenomenon. For DFPT, spontaneous polarization is assumed to map onto the center Ti atom of a unit cell; thereby, the spontaneous polarization of bulk BaTiO_3_ can be calculated using Equation (6) [46]:

(6) $$P_{k}=\frac{e}{\Omega_{k}}[(\mu_{k}^{Ti}-\mu_{k0})•Z^{*}{\;}_{Ti}+\frac{1}{2}\sum_{i=1}^{6}\left(\mu_{ki}^{O}-\mu_{k0}\right)•Z^{*}{\;}_{O}+\frac{1}{8}\sum_{i=1}^{8}\left(\mu_{kj}^{Ba}-\mu_{k0}\right)•Z^{*}{\;}_{Ba}]$$

where *k* denotes the Ti-centered unit cell; ***u****_k_*_0_ indicates the central position of the cell; *k_i_* and *k_j_* denote the oxygen and barium atoms; ***u_k_^Ti^***, ***u_k_^O^***, and ***u_k_^Ba^*** are the atomic positions of elemental Ti, O, and Ba; the (***u_k_^Ti^***−***u_k0_***), (***u_k_^O^***−***u_k_*_0_**), and (***u_k_^Ba^***−***u_k_*_0_**) are the distances of the Ti, O, and Ba atoms from the center of the BaTiO_3_ unit cell; **Ω_k_** is the volume; and Z* is the Born effective charge tensor.

**Figure 6:**
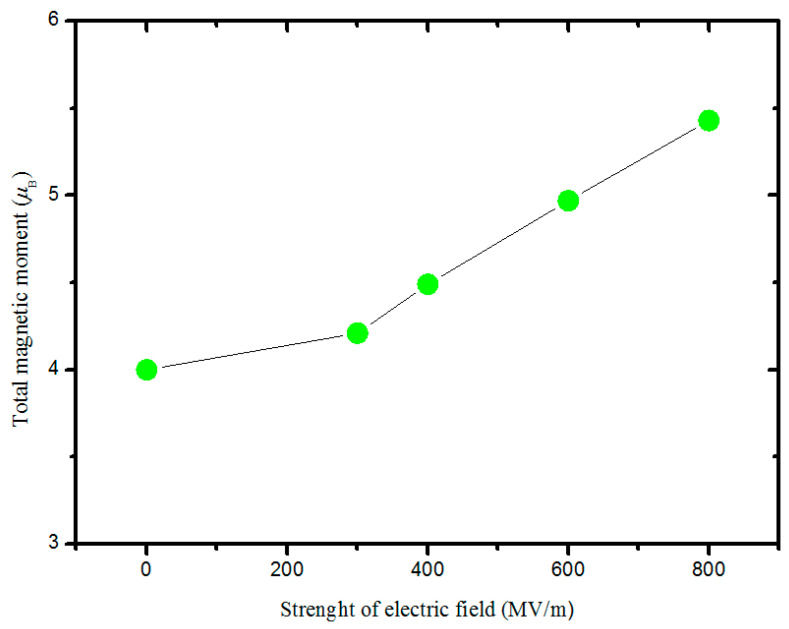
Total magnetic moment as a function of the electric field in a two-dimensional BaTiO_3_ (100) ultrathin film with Co occupying two neighboring Ti sites in the [001] direction. Its dimensions are 2 × 3 × 4.

**Figure 7:**
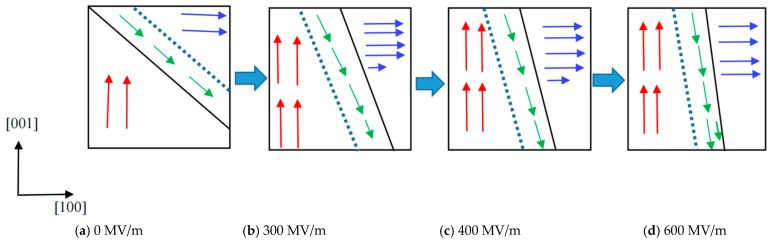
Schematic of the magnetic domain turned in a two-dimensional BaTiO_3_ (001) ultrathin film with Co in the neighboring Ti sites as a function of the electric field applied in the [100] direction; the arrows represent the magnetic moments; blue is parallel to the [010] direction, red aligns with the [100] direction, and green describes an angulation in the [100], [010], and [001] directions.

The spontaneous polarization of BaTiO_3_ ultrathin films with one Co in the Ti site and a 4 × 2 × 2 dimension (atomic concentration of Co is 1.04%) was calculated to be 0.26 μC/cm^2^, 0.28 μC/cm^2^, and 0.71 μC/cm^2^, respectively, corresponding to the [100], [010], and [001] directions. It can be seen that the spontaneous polarization in the [001] direction is the highest and that the [100] and [010] directions are the lowest. Theoretically, the spontaneous polarization in the [100] direction should equal the [010] direction, and calculation error may cause this slight difference. Therefore, to obtain an ME coupling, a simulation model of a BaTiO_3_ (100) ultrathin film was built, and an electric field was applied in the [100] direction. The vacuum layer was set at ~32 Å in the [100] direction. The field’s strength ranged from 0 MV/m to 800 MV/m. It is worth stating that the magnetic behavior is similar to that of the (001) configuration in the free-standing state, as discussed above. For example, it was found that the total magnetic moment changes slightly and stays at nearly 1.00 μ_B_ in the case of one Co substituting Ti in the supercell. Hence, the neighboring Ti model was employed to study ME coupling. Moreover, the total magnetic moment is around 4.00 μ_B_ when the electric field is lower than 100 MV/m; with increasing electric field strength, the total magnetic remains around 2.00 μ_B_. To deduce the evolution, the optimized geometric structure was analyzed. In the doped unit cell, the neighboring substitution of Ti in the [100] direction causes the ferroelectric phase to shift back to the paraelectric state in the [001] direction, which reduces spontaneous polarization. As a result, the spontaneous polarization in the doped cell is slightly altered under an electric field because it is approximately zero and exhibits a constant magnetic moment. To induce a displacement between Co and O, the neighboring Ti in the [001] direction was replaced, and the dimension of the simulation model was set as 2 × 3 × 4, comprising 144 atoms, as shown in Figure 4. The relationship between the electric field and magnetic moment is shown in Figure 6; clearly, with an enhanced electric field, the total magnetic moment increases. When the electric field is enhanced from 0 MV/m to 800 MV/m, the total magnetic moment increases from 4.00 μ_B_ to 5.43 μ_B_. In the case of electric fields of 0 MV/m, 300 MV/m, 400 MV/m, 600 MV/m, and 800 MV/m, the total magnetic moment was calculated at 4.00 μ_B_, 4.21 μ_B_, 4.49 μ_B_, 4.97 μ_B_*,* and 5.43 μ_B_*,* respectively, showing that the magnetic behavior can be controlled using an electric field. It is well known that when BaTiO_3_ is placed in an electric field, the ferroelectric ultrathin film will become polarized, and all spontaneous polarization will realign to the direction of the electric field, which means that the spontaneous polarization in the [010] and [001] directions will decrease. Meanwhile, the magnetic domain turns and leads to a change in the total magnetic moment. To shed light on the evolution of magnetic behavior under an electric field, the partial magnetic moments in the [100], [010], and [001] directions were estimated, as listed in Table 2, and they were calculated to be 2.00 μ_B_, 2.00 μ_B_, and 2.00 μ_B_, respectively, without any external fields. When a continuously increasing electric field is applied, the partial magnetic moments in the [010] and [001] directions are nearly around 4.00 μ_B_, and that in the [100] direction increases. Clearly, the partial magnetic moment is enhanced with the application of an electric field, showing an ME coupling, but it is difficult to adjust in the [010] and [001] directions. Interestingly, the partial magnetic moment in the [100] direction is nearly equal to the total magnetic moment. It can be illustrated that the ME coupling is mainly contributed by the partial magnetic moment in the [100] direction. The schematic of the evolution of magnetic domains under electric fields is provided in Figure 7. In the configuration, there exist other magnetic domains in the directions except for the [100], [010], and [001] directions. It not only shows an enhancement in the magnetic moment but also a movement of the magnetic domain when an electric field is applied. With an enhanced electric field, the magnetic moments in other directions are turned to align with the [001] direction. As a result, the total magnetic moment is lower than the composition of the magnetic moments in the [100], [010], and [001] directions.

### 3.3. Magnetoelectric Coupling

In our study, the total magnetic moment increases with an enhanced electric field, showing an ability to select the orientation of the magnetic domain using an external electric field. Thus, an evaluation on ME coupling was carried out, which is defined as an induced magnetization ***M*** with an external electric field ***E*** [47,48]:

(7) ΔM=αΔE

where Δ*M* and Δ*E* represent changes in the total magnetic moment and strength of the electric field, and *α* is the ME coupling constant.

**Figure 8:**
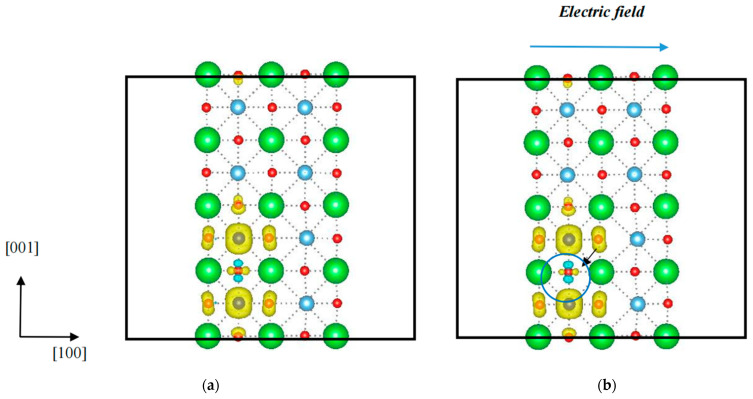
Spin-polarized charge densities of two-dimensional BaTiO_3_ (010) doped with Co in the Ti sites (**a**) without and (**b**) with an 800 MV/m electric field. The dotted lines denotes the chemical bonds between atoms.

According to Equation (6), the *α* values were calculated at 0.000007 μ_B_/MVm, 0.000027 μ_B_/MVm, 0.0000185 μ_B_/MVm, and 0.000029 μ_B_/MVm, respectively, corresponding to electric fields from 0 MV/m to 300 MV/m, 300 MV/m to 500 MV/m, 500 MV/m to 800 MV/m, and 300 MV/m to 800 MV/m, showing a high ME coupling constant. Clearly, when the electric field is 300 MV/m, the *α* value has the minimum value because when the applied electric field increases slightly over the threshold value ***E_c_*** (the critical electric field to turn the magnetic domain), the change induced in the total magnetic moment is small; as a result, the corresponding ME coupling constant is much lower. Thereby, calculated data corresponding to an electric field higher than 300 MV/m were adopted to evaluate the ME coupling constant. Moreover, the *α* value was estimated at ~0.0000248 μ_B_/MVm or ~449.3 mV/cmOe, suggesting a superior multiferroic property at room temperature, relative to the Fe case [14]. When an electric field was applied, the spontaneous polarization turned toward the direction of the electric field, which was confirmed in our previous experimental study [5] It indicated that a strain was induced. However, when a strain was applied to a two-dimensional BaTiO_3_, only a slight change was observed. For instance, under a 4% compressive strain, the total magnetic moment remained around 4.00 μ_B_; however, in the case of an electric field, the total magnetic moment varied owing to a redistribution of spin electrons. Figure 8 shows the spin-polarized charge densities of a two-dimensional BaTiO_3_ ultrathin film without and with an electric field. A distinct change appeared in the spin-polarized charge density in the blue circle when an electric field was applied. 

In summary, when strict stoichiometry and appropriate technology were selected to grow the Co-doped BaTiO_3_ epitaxial structure, a magnetic signal can be captured using experimental technology. Otherwise, interstitial Co will form, which will lead to the disappearance of magnetic signals. Furthermore, the O vacancy was confirmed to be difficult to form, indicating that the interstitial Co mainly causes the demagnetization. The neighboring Ti substitution in the [001] direction induced a high-performing multiferroic property in two-dimensional BaTiO_3_ (100) ultrathin films, and the corresponding ME coupling constant reached ~449.3 mV/cmOe, showing that BaTiO_3_ ultrathin films may be a good candidate for single-phase multiferroics to be applied in next-generation multifunctional devices.

## 4. Conclusions

In this study, a first-principles approach was utilized to study the magnetic behavior of two-dimensional BaTiO_3_ ultrathin films doped with Co under an electric field. Taking the structural stability into account, only the sites of Co occupying Ti vacancies, Co dissolving into interstitial sites, and Co in the Ti vacancy and interstitial space were considered. As calculations showed, formation energies were negative, indicating that all configurations were energetically stable. And the formation energy of Co in the interstitial space was lower than that of Co in the vacancy at −7.77 eV and −3.26 eV, respectively. The enthalpy of the formation of two-dimensional BaTiO_3_ doped with Co also showed structural stability. Besides that, the trapping energy suggested that the interstitial Co atom readily formed, which demagnetized the Co-doped BaTiO_3_ ultrathin film. And the maximum number of interstitial Co was only one. When the interstitial and vacant Co existed in a 1:1 ratio, the configuration behaved in a nonmagnetic manner. In contrast, either interstitial Co or vacant Co could induce magnetic moments in BaTiO_3_ ultrathin films. Moreover, the total magnetic moment was in a linear relationship with the Co content in the case of dopants with a distance of at least two unit-cell intervals from each other. Among them, only the neighboring Ti substitution exhibited a coupling between Co, which led to the formation of spontaneous polarization. Furthermore, the magnetic behavior of two-dimensional BaTiO_3_ (100) and (001) ultrathin films was similar in their free-standing states. 

To apply an electric field to two-dimensional BaTiO_3_, the corresponding semiconductor characteristic was studied. It was shown that the band gap disappeared when the atomic concentration of Co reached 2.08%. Hence, a supercell with a 2 × 3 × 4 dimension was built, and its electronic density of states demonstrated that the two-dimensional BaTiO_3_ behaved in the manner of a semiconductor. However, upon employing an electric field in the [001] direction, it was observed that the total magnetic moment was nearly nonadjustable in the case of a two-dimensional BaTiO_3_ (001) ultrathin film, i.e., when the electric field increased, the total magnetic moment changed slightly. It was confirmed to be caused by the low spontaneous polarizations of 0.26 μC/cm^2^ and 0.28 μC/cm^2^ in the [100] and [010] directions; thus, the (100) configuration was adopted to study the electric control of magnetic behavior. The calculations showed that with an enhanced electric field from 0 MV/m to 800 MV/m, the total magnetic moment increased from 4.00 *μ_B_* to 5.43 μ_B_, in the order of 4.00 μ_B_, 4.21 μ_B_, 4.49 μ_B_, 4.97 μ_B_*,* and 5.43 μ_B_, respectively, corresponding to electric fields of 0 MV/m, 300 MV/m, 400 MV/m, 600 MV/m, and 800 MV/m. Meanwhile, the partial magnetic moment in the [100] direction increased; in the [010] and [001] directions, it increased to ~4.00 μ_B_ when the electric field reached 300 MV/m, then changed slightly. And we revealed that there were other magnetic domains in the configuration, excluding those in the [100], [010], and [001] directions. This indicates that the magnetic domain in the other directions turns away to align with the [001] direction. Furthermore, the magnetoelectric coupling constant was calculated to be ~449.3 mV/cmOe, showing a high-performance multiferroic property.

## Figures and Tables

**Table 1:** Formation energy of two-dimensional BaTiO_3_ with Co occupying different sites.

Site	Formation Energy (eV)
Vacancy + Co	−3.26
Interstitial site	−7.77
Interstitial site with a 1.5-unit-cell distance to vacancy	−0.64
Interstitial site nearest to vacancy	−2.08

**Table 2:** Partial magnetic moments in two-dimensional BaTiO_3_ with Co occupying Ti sites in different directions under electric fields.

Electric Field (MV/m)	Partial Magnetic Moment (μ_B_)		
	[100]	[010]	[001]
0	2.00	2.00	2.00
300	4.21	4.02	4.05
400	4.48	3.98	4.01
600	4.91	3.89	4.00
800	5.43	4.06	3.98

## Data Availability

The data that support the findings of this study are available from the corresponding authors upon reasonable request.
